# Engagement With a Trauma Recovery Internet Intervention Explained With the Health Action Process Approach (HAPA): Longitudinal Study

**DOI:** 10.2196/mental.9449

**Published:** 2018-04-10

**Authors:** Carolyn M Yeager, Kotaro Shoji, Aleksandra Luszczynska, Charles C Benight

**Affiliations:** ^1^ Department of Psychology University of Colorado Colorado Springs Colorado Springs, CO United States; ^2^ Trauma Health and Hazards Center University of Colorado Colorado Springs Colorado Springs, CO United States; ^3^ SWPS University of Social Sciences & Humanities Wroclaw Poland

**Keywords:** electronic health (eHealth), engagement, trauma, stress disorders, post-traumatic, PTSD, Health Action Process Approach (HAPA), outcome expectations, internet, digital health intervention

## Abstract

**Background:**

There has been a growing trend in the delivery of mental health treatment via technology (ie, electronic health, eHealth). However, engagement with eHealth interventions is a concern, and theoretically based research in this area is sparse. Factors that influence engagement are poorly understood, especially in trauma survivors with symptoms of posttraumatic stress.

**Objective:**

The aim of this study was to examine engagement with a trauma recovery eHealth intervention using the Health Action Process Approach theoretical model. Outcome expectancy, perceived need, pretreatment self-efficacy, and trauma symptoms influence the formation of intentions (motivational phase), followed by planning, which mediates the translation of intentions into engagement (volitional phase). We hypothesized the mediational effect of planning would be moderated by level of treatment self-efficacy.

**Methods:**

Trauma survivors from around the United States used the eHealth intervention for 2 weeks. We collected baseline demographic, social cognitive predictors, and distress symptoms and measured engagement subjectively and objectively throughout the intervention.

**Results:**

The motivational phase model explained 48% of the variance, and outcome expectations (beta=.36), perceived need (beta=.32), pretreatment self-efficacy (beta=.13), and trauma symptoms (beta=.21) were significant predictors of intention (N=440). In the volitional phase, results of the moderated mediation model indicated for low levels of treatment self-efficacy, planning mediated the effects of intention on levels of engagement (B=0.89, 95% CI 0.143-2.605; N=115).

**Conclusions:**

Though many factors can affect engagement, these results offer a theoretical framework for understanding engagement with an eHealth intervention. This study highlighted the importance of perceived need, outcome expectations, self-efficacy, and baseline distress symptoms in the formation of intentions to use the intervention. For those low in treatment self-efficacy, planning may play an important role in the translation of intentions into engagement. Results of this study may help bring some clarification to the question of what makes eHealth interventions work.

## Introduction

### Background

There has been a growing trend in the delivery of mental health treatment over the internet [1]. Results of a recent survey found that 87% of American adults now use the internet, and of those users, over 80% look online for health-related material [2]. Likewise, the numbers of online psychotherapeutic interventions (ie, electronic health, eHealth) have also increased [3]. This increase may be because of several advantages offered by eHealth interventions, such as reduced stigma, costs, and increased autonomy, anonymity, and accessibility [4]. However, engagement with eHealth interventions is a concern. Theoretically based research in this area is sparse [5] despite consistent evidence suggesting engagement is essential for optimizing outcomes [6]. This study examined engagement with a trauma recovery eHealth intervention using a theoretical model explaining how and why people engage.

Exposure to potentially traumatic events in adult US populations is widespread [7]. A significant number of those exposed will develop posttraumatic stress disorder (PTSD) along with depression, anxiety, and substance use disorders [8]. Finding ways to treat traumatized populations to reduce the associated medical, psychological, and social costs is essential [9]. There is a growing concern that those with more persistent mental health issues following trauma are reluctant to seek treatment [10]. Significant barriers to treatment include logistical, geographical, financial, stigma, and other attitudinal challenges [11]. One promising approach to overcoming these barriers is the provision of mental health services via technology that can be readily standardized for broad dissemination of evidence-based care.

Ample research has shown eHealth interventions are effective in decreasing distress symptoms in trauma survivors [12-15]. However, limited participation and high attrition rates are common [16,17]. As the amount of exposure to an intervention is strongly linked to behavioral outcomes [18], understanding the factors that influence engagement is a major step in improving their effectiveness [19].

### Study Aim

Our study aimed to examine the utility of using a single theoretical model, the Health Action Process Approach (HAPA) [20], to evaluate differential predictors of eHealth engagement for trauma recovery. The HAPA examines stages of behavior change and considers psychological factors and self-regulatory strategies to model both direct and indirect pathways of engagement, irrespective of the technological features of the intervention.

### Electronic Health and Engagement

The term engagement has been used in a variety of ways, making it challenging to synthesize consistent models and measures. Generally, engagement is described as efforts by a user to start and continue with an intervention and encapsulates objective and subjective experiences [21]. However, this definition of engagement is not consistently observed across the literature. For the purposes of our study, we define engagement objectively and subjectively as a measure of how participants interact with the eHealth intervention, including how long and how often the intervention is used. This definition of engagement is sometimes referred to as the micro level of engagement [21]. Engagement is different from adherence, which refers to using the intervention as intended. Attrition occurs when an individual drops out of the intervention before completion (ie, nonadherence). Attrition from open access nontracked websites can be very high, with as few as 1% of users completing a full course of online therapy [22]. Attrition from traumatic stress–related interventions can be especially problematic [23]. Studies have found attrition rates ranging from 36% to 78% [24,25]. As a result, the degree of engagement (or lack thereof) can have a significant effect on key outcomes and impact on quality of life.

Predictors of engagement with eHealth interventions more generally and trauma programs more specifically have not been studied in a systematic, theoretically based way [6]. Previous a-theoretical approaches have investigated potential predictors of engagement with mixed findings. These studies focused on how user characteristics such as demographics [26], health problems, and social factors [27] affect engagement. However, meta-analytic findings suggested limited evidence for any specific individual characteristic that may influence engagement with eHealth interventions [28].

Other researchers have focused on effects of the technical design aspects on engagement [29]. These components include varying levels of interactivity [30], gamification [31], tailoring [32], modality (mobile vs Web), and software sophistication [33]. These ever-evolving features can be combined in countless ways, making engagement research difficult to generalize across interventions.

Researchers from areas beyond trauma (eg, health and illness issues) have applied theoretical frameworks to explain eHealth engagement. The Technology and Acceptance Model has been used to explore intentions to engage with information and communication technologies among health care providers [34]. This model examined perceived usefulness and ease of use but failed to consider perceptions of need, self-efficacy, and symptom severity. Other approaches combined multiple theoretical models to address different components of eHealth engagement separately, such as health service utilization and technology acceptance theories [35], but do not consider all components simultaneously. Mâsse [36] used the theory of planned behavior and self-determination theory to examine engagement with an eHealth obesity intervention and found intentions did not directly predict engagement. One possible explanation may be that, unlike the HAPA, these theories did not consider indirect pathways through which intentions are translated into engagement. Recently, Kok [19] examined nonadherence to phobia interventions and suggested that patient expectations and baseline symptom severity could affect adherence to eHealth interventions.

### Health Action Process Approach Model

**Figure 1:**
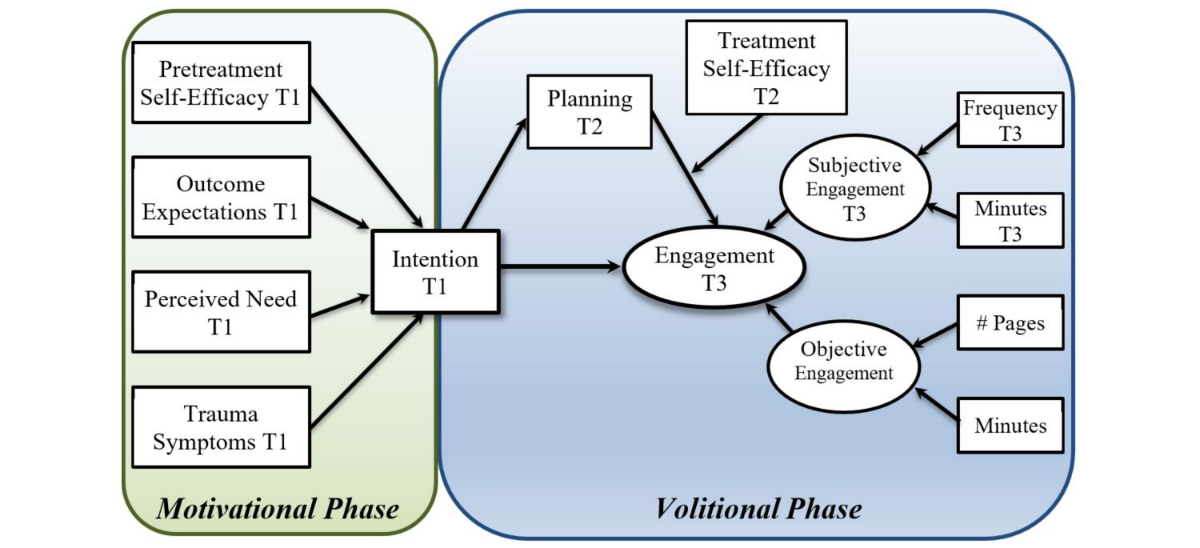
Longitudinal revised Health Action Process Approach (HAPA) research model. In the motivational phase, pretreatment self-efficacy, outcome expectations, perceived need, and trauma symptoms are predicted to have a significant positive effect on the formation of intentions. In the volitional phase, intentions are translated into engagement, mediated by planning and moderated by levels of treatment self-efficacy. Engagement is a latent construct consisting of both subjective (estimated frequency and duration) and objective measures. Objective engagement is continuously measured by the electronic health (eHealth) intervention.

The HAPA [20] is an approach developed to predict engagement in health behavior. The model has good predictive validity across a variety of preventative health behaviors, including physical exercise [37,38], nutrition [39], and cancer screening [40]. Our study is a novel application of the HAPA (Figure 1) to investigate engagement with a trauma recovery eHealth intervention. HAPA addresses both motivational and volitional processes, with different patterns of social-cognitive predictors emerging in respective phases. These patterns, as they relate to eHealth engagement with a trauma recovery intervention, were explored in this study.

#### Motivational Phase

The HAPA motivational phase is typically characterized by awareness of risk, outcome expectancies, and perceived task self-efficacy (ie, pretreatment self-efficacy). For our eHealth intervention, positive outcome expectancies may refer to the ability to cope with posttraumatic distress. Pretreatment self-efficacy reflects beliefs about the ability to initiate eHealth engagement [41]. Individuals high in pretreatment self-efficacy imagine success and are more likely to adopt a new behavior [39].

Besides self-efficacy and outcome expectations, the role of other motivational variables such as perceived need and posttraumatic symptoms may be considered in the motivational phase of the HAPA. Perceived need [39] is defined as one’s perception of needing an intervention for trauma-related symptoms such as anxiety, depression, and other PTSD symptoms. Perceived need may lead to deliberations about behavior change [42]. The construct of perceived need for a coping support intervention may be considered conceptually similar to a construct of perceived risk [20]. Furthermore, the degree of distress or PTSD symptoms [43] may affect the perceived capability to manage distress or utilize available resources following a traumatic event [44]. Research found baseline PTSD symptoms positively related to engagement [27]. However, the relationship between baseline mental health symptoms and treatment engagement indicators such as attrition is unclear, where higher attrition is associated with higher symptoms in some studies [45,46] vs lower baseline PTSD symptoms in others [47]. It is possible that baseline symptom severity can serve as an index of perceived need or as a barrier to participation.

#### Volitional Phase

After intention has been formed in the motivational stage of the HAPA, an individual enters the volitional stage where self-regulation skills such as planning and treatment self-efficacy prompt behavior enactment [20,48]. Planning specifies when, where, and how a behavior will be implemented [49]. Planning may refer to emerging barriers that would prevent one from acting as planned [50]. Treatment self-efficacy refers to the perceived ability to maintain a new behavior and cope with arising barriers. Adhering to a trauma recovery eHealth intervention may turn out to be far more challenging than expected, but a self-efficacious person should respond confidently and develop better strategies for responding to arising difficulties [40].

### Engagement

The primary outcome of this study was engagement with a trauma recovery eHealth intervention. A major challenge in the study of engagement is the lack of a shared definition and conceptualization of user engagement [21]. Historically, behavior-based metrics such as page views and time online have been used as indicators of engagement [51]. As Danaher [52] noted, “a key ingredient in determining the impact of any Web-based behavior change program is the extent to which participants are exposed to the program.” However, intervention exposure alone fails to capture the experiential aspects of engagement. A recent systematic review concluded that a valid and reliable conceptualization of engagement needs to consider objective and subjective measures that include behavioral and experiential dimensions of eHealth intervention interactions [53].

High quality, objectively measurable information on engagement can be acquired from page logs, time on site, and other indicators of treatment exposure. These objectively measurable metrics were included in our study. Our study also included self-report measures of engagement to capture subjective perceptions of usage.

### Study Hypotheses

Using the HAPA as a model guiding the relationships between the study variables (Figure 1), we hypothesized time 1 (T1) pretreatment self-efficacy, outcome expectancy, perceived need, and PTSD symptoms would be positively related to the formation of intentions to engage at T1 (the motivational hypothesis). Once intentions were formed, time 2 (T2) planning was hypothesized to mediate the translation of T1 intentions into eHealth intervention engagement at time 3 (T3), moderated by the level of T2 treatment self-efficacy (the volitional hypothesis).

## Methods

### Participants

To increase external validity, participants were recruited from the Trauma, Health, and Hazards Center trauma registry (15/440, 3.4%), national domestic violence advocate and rape crisis center registries (64/440, 14.5%), the national development and research institute list servers (56/440, 12.7%), social media (19/440, 4.3%), and the University of Colorado at Colorado Springs (UCCS) student population (286/440, 65.0%) from May 2015 to October 2016. All participants included in the study had directly experienced one or more traumatic events as measured by the Life Events Checklist [54], were 18 years or older, had a private area to access the internet, and spoke English. Table 1 displays the demographic information. Of the 626 who completed the T1 survey, 440 participants qualified for the study (mean age 25.57 years; SD 11.02; 337/440, 76.6 % female; 66/440, 15% Hispanic). Of those who qualified, 186 created an account on the website, 161 participated in the T2 survey (mean age 28.11 years; SD 13.31; 128/161, 79.5% female), and 115 participated in the T3 survey (mean age 28.49 years; SD 12.98; 94/115, 81.7% female). Those failing to access the intervention (ie, nonuse attrition) were not considered for T3 analyses.

All participants who met criteria at T1 (N=440) reported that they were directly exposed to one or more traumatic events either through experiencing or witnessing the event, including physical assault (248/440, 56.4%), transportation accidents (300/440, 68.3%), unwanted sexual contact (219/440, 49.9%), sexual assault (158/440, 35.9%), life-threatening illness or injury (225/440, 51.1%), fire or explosion (167/440, 38%), natural disasters (175/440, 39.7%), sudden violent death of someone close (109/440, 24.9%), serious accidents (150/440, 34.2%), severe human suffering (136/440, 30.9%), toxic exposure (68/440, 15.5%), military combat (37/440, 8.5%), and other traumatic events (258/440, 58.6%). Participants experienced the same traumatic event with varying frequency, ranging from 56.4% (248/440) who experienced the event once, to 9.1% (40/440) who experienced the event at least 14 times.

### Procedure and Study Design

**Figure 2:**
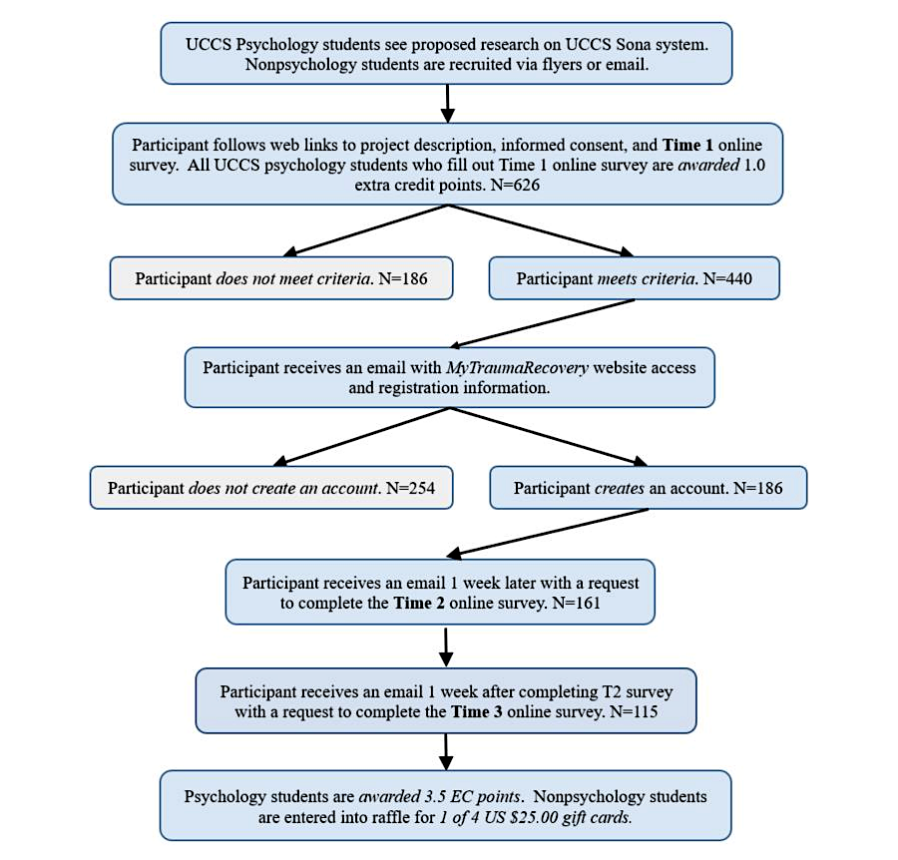
Participant flowchart.

The UCCS Institutional Review Board approved the study. UCCS psychology students signed up for this study via the Sona online system, and nonstudents were contacted via email or a flyer. All participants were provided a brief statement explaining the procedure and purpose of the study along with a link to the T1 survey on Qualtrics. Figure 2 illustrates the participant flowchart. After participants read the online informed consent form and clicked the “I accept” button, they completed the T1 questionnaire. Participants who completed the T1 survey and met the inclusion criteria were given access to the eHealth intervention via email. The email provided participants with the website address and instructions on how to create a user account, log in to the site, and directed them to use the site as much as they would like over the next 2 weeks.

One week after qualifying for the study, participants were sent an email asking them to complete the short T2 survey. One week after finishing the T2 survey, participants were prompted by email to take the T3 survey on Qualtrics. After finishing the final survey, participants were debriefed, and UCCS psychology students received additional extra credit. Nonpsychology students were entered into a raffle for one of four US $25.00 gift cards. Local and national mental health resources were provided to all participants after the study.

### Measures

The following measures incorporated variables in the motivation and volitional phases of the HAPA model (see Figure 1). The motivational components were available at T1 and included pretreatment self-efficacy, outcome expectations, perceived need, trauma symptoms, and intention. The volitional phase components were assessed at T2 and T3 and included planning, treatment self-efficacy, and subjective engagement. Objective engagement levels were tracked and recorded automatically by the intervention throughout the study.

In addition to the HAPA variables, participant website satisfaction was also measured. All measures except trauma symptoms were developed for this study, as there were no measures available to assess these constructs. Their psychometric properties are shown in Table 2.

#### Motivation Model Measures

##### Pretreatment Self-Efficacy (Time 1)

Pretreatment self-efficacy was measured by eight questions that began with the sentence stem “I am confident that I can start using an eHealth intervention in the next two weeks...” The sentence stem was followed by items representing technological and coping related barriers such as “even if I am uncomfortable using the internet” or “even if I am having difficulty handling all the things I have to do.” Participants responded on a 5-point scale ranging from not at all confident to very confident.

**Table 1:** Descriptive statistics for demographics for time 1 (baseline), time 2 (one week after baseline), and time 3 (two weeks after baseline). Some percentages do not add up to 100% because of missing data.

Measure		Time 1 (N=440)	Time 2 (N=161)	Time 3 (N=115)
Mean age in years (SD)		25.57 (11.02)	28.11 (13.31)	28.49 (12.98)
Age range in years		18-80	18-80	18-78
**Gender, n (%)**				
	Female	337 (76.6)	128 (79.5)	94 (81.7)
	Male	101 (23.0)	33 (20.5)	21 (18.3)
	Other	2 (0.5)	0 (0.0)	0 (0.0)
**Intimate relationship, n (%)**				
	Single^a^	192 (43.6)	38 (39.2)	43 (37.4)
	Committed^b^	213 (48.4)	50 (51.5)	60 (52.2)
	Other	35 (8.0)	9 (9.3)	12 (10.4)
**Highest education, n (%)**				
	High school	280 (63.3)	43 (44.3)	60 (52.2)
	Associates degree	95 (21.6)	24 (24.7)	30 (26.1)
	Bachelor’s degree	45 (10.2)	20 (20.6)	18 (15.7)
	Graduate degree	18 (4.1)	10 (10.3)	07 (6.1)
**Employment, n (%)**				
	None	117 (26.6)	22 (22.7)	24 (20.9)
	Part-time	196 (44.5)	34 (35.1)	52 (45.2)
	Full-time	116 (26.4)	33 (34.0)	34 (29.6)
	Retired	10 (2.3)	8 (8.2)	05 (4.3)
**Income (USD), n (%)**				
	$0-$25,000	186 (42.3)	42 (43.3)	46 (40.0)
	$25,001-$70,000	143 (32.5)	34 (35.1)	42 (36.5)
	$70,001-$100,000	58 (13.2)	10 (10.3)	13 (11.3)
	>$100,000	50 (11.4)	10 (10.3)	12 (10.4)
**Mental health, n (%)**				
	Treatment (current)	71 (16.1)	17 (17.5)	22 (19.1)
	Treatment (past year)	32 (7.3)	9 (9.3)	7 (6.1)
	Treatment (lifetime)	183 (41.6)	51 (52.6)	57 (49.6)
**Frequency of traumatic event, n (%)**				
	1 time	248 (56.4)	80 (49.7)	53 (46.1)
	2-13 times	129 (29.3)	51 (31.7)	36 (31.3)
	>14 times	40 (9.1)	20 (12.4)	17 (14.8)

^a^Includes widowed or divorced.

^b^Includes married couples and couples in a committed relationship.

**Table 2:** Number of items, scoring range, and Cronbach alpha for time 1 (N=440), time 2 (N=161), and time 3 (N=115) measures. PCL-5: Posttraumatic Stress Disorder Checklist for Diagnostic and Statistical Manual of Mental Disorders, Fifth Edition.

Scale	Number of items	Scoring range	Cronbach alpha T1	Cronbach alpha T2	Cronbach alpha T3
1. PCL-5	20	0-80	.95	—^a^	.95
2. Outcome expectations	10	10-50	.85	.83	.85
3. Pretreatment self- efficacy	8	8-40	.95	—	—
4. Perceived need	6	6-30	.92	—	—
5. Intention	5	5-25	.88	—	—
6. Treatment self-efficacy	8	8-40	—	.96	.94
7. Planning	4	4-20	—	.80	.79
8. Engagement (subjective)	10	—	—	—	.86

^a^Not measured at respective time point.

##### Outcome Expectancies (Time 1, Time 2, and Time 3)

Both positive (pros) and negative (cons) outcome expectancies were assessed with 10 questions that started with the sentence stem “If I use the eHealth intervention on a regular basis I expect that...” followed by the items measuring possible pros and cons. Example pros and cons include “it will help me to relax more” or “it will not make any difference in how I feel.” Cons were reverse scored, and the total score was computed by adding the answers to all items.

##### Perceived Need (Time 1)

Perceived need was measured with six responses to the following statement: “Please indicate your perception of how much you believe you need an intervention for the following issues.” Issues pertain to dealing with the trauma such as “to feel normal again?” and “to be able to manage distressing dreams or images about the traumatic experience.” Participants responded on a 5-point scale ranging from strongly disagree to strongly agree.

##### Posttraumatic Stress Disorder Checklist for Diagnostic and Statistical Manual of Mental Disorders, Fifth Edition (Time 1 and Time 3)

The PTSD Checklist for Diagnostic and Statistical Manual of Mental Disorders, Fifth Edition (DSM-5; PCL-5) was used to measure the distress symptoms associated with trauma. The PCL-5 is a 20-item self-report measure that assesses the 20 DSM-5 symptoms of PTSD [55]. PCL-5 has shown strong internal consistency (alpha=.94) and test-retest reliability (*r*=.82) [56]. The PCL-5 was scored using a total symptom severity score (range, 0-80) by summing the scores for each of the 20 items.

##### Intention (Time 1)

Behavioral intentions are the perceived likelihood to act in a certain way, and for this study, they comprised a person’s motivation toward using an eHealth intervention. Intention to perform a behavior should be measured the same way as assessing the behavior itself [50]. Intention to use the eHealth intervention was measured by five questions that began with the sentence stem “During the next two weeks I intend to use the eHealth intervention to help me...” Example questions include “to learn relaxation skills” or “fight negative thinking.” Responses ranged from strongly disagree to strongly agree.

#### Volitional Model Measures

##### Treatment Self-Efficacy (Time 2 and Time 3)

Treatment self-efficacy was measured by eight questions that began with the sentence stem “I am confident I could continue to use an eHealth intervention over the next two weeks...” followed by items measuring treatment self-efficacy related technology and trauma coping self-efficacy. Example items include “even if I do not like it initially” and “even if it brings up difficult memories.” Participants responded on a 5-point scale ranging from not at all confident to very confident.

##### Planning (Time 2 and Time 3)

Individuals were asked if they had made a plan or schedule for using the eHealth intervention. For those who planned, details of their plan were measured by four questions that began with the sentence stem “My plan included...” followed by questions regarding when, where, what, and how often they would use the intervention. Example questions include “how often I would use the eHealth intervention” and “what modules of the eHealth intervention I would use.” Responses ranged from strongly disagree to strongly agree.

##### Engagement (Time 3)

Engagement was measured both subjectively and objectively. Subjective measures included questions regarding frequency and duration assessed at T3. Frequency was assessed with five questions that began with the sentence stem “How often did you use the following eHealth intervention modules...” followed by a list of five modules (unhelpful ways of coping, relaxation, social support, self-talk, trauma triggers, and memories). Answers were rated on a 6-point scale ranging from 1 (“never”) to 6 (“more than once a day”). Duration was measured by the total estimated usage (in minutes) of the five modules. Objective measures consisted of automatically recorded data that quantified the frequency (number of pages visited) and duration (total number of minutes logged in) of intervention usage [18,52]. Inactive minutes were not included in the objective duration calculation. Participants were deemed inactive when their login time exceeded 15 min without any corresponding page activity. Subjective and objective variables were combined as observed variables loading a respective latent variable to represent overall engagement (see Figure 1).

### Electronic Health Intervention—My Trauma Recovery

My Trauma Recovery (MTR) is a self-guided, theoretically based, interactive internet application with no interaction with a therapist. MTR is based mainly on social cognitive theory [57], where individuals are viewed as proactive agents who can choose their environments, find beneficial social networks, and engage in self-management behaviors that allow them to both initiate and maintain long-term recovery [13].

The intervention focuses on increasing an individual’s ability to cope with trauma via six self-directed modules: (1) unhelpful ways of coping, (2) relaxation, (3) social support, (4) self-talk, (5) trauma triggers and memories, and (6) seeking professional help. The first five modules were included in the subjective engagement measure. A self-test provides users the opportunity to gain feedback on their current emotional distress and provides graphs that depict their assessment results. Throughout the six modules, the site utilizes mastery experiences, vicarious success modeling, verbal persuasion, and tools to monitor and regulate internal distress to increase coping self-efficacy through interactive, tailored experiences. Individuals can use any of modules as often as needed and can assess their progress over time. On the basis of their assessments, suggestions are made to maximize trauma coping self-efficacy gains. Completing all the MTR modules requires approximately 2 hours. However, users generally do not finish all the modules in a single sitting. Additionally, the interactive components of the website offer opportunity to revisit the different modules over time (eg, triggers management or relaxation). Therefore, we allowed participants access to the intervention for 2 weeks to provide ample opportunity to explore all components of MTR.

MTR has received initial support for its effectiveness in reducing symptoms in two separate randomized clinical trials [58,59]. The first randomized clinical trial with disaster survivors following hurricane Ike demonstrated that the MTR website participants improved significantly on worry with little change for the comparison information only or waitlist groups. A marginal effect for depression was also identified [58]. The website was also evaluated in a randomized clinical trial with two populations in China where significant positive effects were also found [59]. Benight and colleagues [13] describe how the website utilizes interactive components (eg, video and audio modeling), question and answer segments, verbal persuasion, and mastery to promote engagement and empowerment.

### Statistical Analysis

Due to the small sample size at T3 (N=115) compared with T1 (N=440), separate analyses were run for the motivational (T1) and volitional (T2, T3) phases. The motivational hypothesis was analyzed with the T1 sample (N=440), and the volitional hypothesis was analyzed with completers only (N=115; see Figure 1). Two participants completed T3 but did not complete T1 or T2. Therefore, they were removed from the final dataset. Additionally, there were two duplicate instances of a participant (did the survey twice) in which case the first instance was used. Data were assessed for outliers, normality, and collinearity. The collinearity tolerance statistic was below .20, and there was no correlation between variables above .90. Therefore, there was no indication of multicollinearity.

The motivational phase hypothesis (Figure 1) was tested via structural equation modeling (SEM) using IBM SPSS Amos v24.0 (IBM Corporation, Armonk, NY, USA) with maximum likelihood estimation. Model fit was examined using the chi-square goodness of fit test, as well as the comparative fit index (CFI) [60], tucker-lewis index (TLI) [61], and root mean square error of approximation (RMSEA) [62]. Cutoff points used for the fit indices were CFI >0.96, TLI >0.95, and RMSEA <0.06.

A moderated mediation analysis was performed using Mplus version 7.4 (Muthén & Muthén, Los Angeles, CA, USA) (Figure 1) to test volitional phase hypothesis. This analysis estimates the indirect effect coefficient for each indirect pathway between the independent variable (intention at T1) and the dependent variable (engagement at T3), accounting for the mediator (planning at T2) and moderator (treatment self-efficacy at T2). In this model, treatment self-efficacy was hypothesized to moderate the translation of plans into action. Engagement was modeled as a latent variable consisting of observed objective and subjective measures.

The bootstrapping method was used to test inferences about the significance of mediation effects when treatment self-efficacy was high (1 SD above the mean) and low (1 SD below the mean), with 5000 bootstrap samples. Bootstrap CIs not including zero indicate a significant indirect effect. The bootstrap approach is considered superior to normal theory-based Sobel test for the significance of the mediation [63]. Results of the analysis are presented as standardized coefficients for each parameter.

## Results

### Preliminary Analysis

The descriptive statistics for the demographic variables are shown in Table 1. Attrition analysis revealed that there were no significant differences between T1 and T3 in sex, χ^2^_1_(N=440)=2.7, *P*=.10, and education, *t*_431_=0.792, *P*=.31. However, there was a significant difference between T1 and T3 groups in age, *t*_438_=−3.34, *P*<.001; baseline PTSD symptoms, *t*_438_=−2.79, *P*=.02; and trauma frequency, *t*_415_=−3.15, *P*=.005; where those who completed T3 were older, had greater symptom severity, and had experienced a greater frequency of trauma. Table 2 shows the internal consistency of each of the measures used in the analyses, indicating that all measures had good to excellent reliability.

Table 3 displays bivariate correlation coefficients, means, and SDs for the HAPA study variables of the motivational and volitional phases. The correlations among motivational phase predictors (T1) for all who met the inclusion criteria (N=440) revealed significant positive correlations among intention and PCL-5 scores, outcome expectations, pretreatment self-efficacy, and perceived need. Correlations for the motivational predictors of those who created an account (N=115; shown below the diagonal line) showed similar patterns except for pretreatment self-efficacy, which no longer showed a significant correlation with intention.

The correlations between the volitional phase predictors (T1, T2, and T3) revealed that intention was significantly positively correlated with planning, treatment self-efficacy, and subjective engagement. Notably, treatment self-efficacy exhibited significant positive medium-sized correlations with all the motivational predictors and most of the volitional phase predictors. Interestingly, only the subjective measures of engagement showed significant positive correlations with intention.

Importantly, paired samples *t* tests indicated a clinically significant decrease in PTSD symptoms [59] for completers with at least subthreshold baseline levels of PTSD symptoms (PCL-5 >20; N=66) between T1 (mean 38, SD 13.11) and T3 (mean 25.56, SD 14.33), *t*_65_=8.48, *P*<.001, *d*=0.48. In addition, outcome expectations significantly increased from T1 (mean 34.68, SD 5.07) to T3 (mean 39.29, SD 5.71), *t*_65_=−6.62, *P*<.001.

**Table 3:** Correlations, means, and SDs of Health Action Process Approach (HAPA) variables for time 1 (N=440), time 2 (N=161), and time 3 (N=115). Correlations in the upper diagonal region for time 1 show values for all participants who met criteria at time 1 (N=440). Correlations in the lower diagonal region for time 1 show values of participants who created an account (N=115). Time 1 was assessed at baseline, time 2 was assessed one week after baseline, and time 3 was two weeks after baseline. PCL-5: Posttraumatic Stress Disorder Checklist for Diagnostic and Statistical Manual of Mental Disorders, Fifth Edition

HAPA variables	Time 1					Time 2		Time 3			
	1	2	3	4	5	6	7	8	9	10	11
1. PCL-5	1.00	.07	.10^a^	.52^c^	.41^c^	.27^b^	.25^b^	.34^b^	.33^b^	.16	.12
2. Outcome expectations	.11	1.00	.34^c^	.37^c^	.54^c^	.12	.36^c^	.15	.15	−.10	.01
3. Pretreatment self-efficacy	.06	.19^a^	1.00	.17^c^	.32^c^	.31^c^	.38^c^	.01	.09	.15	.12
4. Perceived need	.55^b^	.44^b^	−.05	1.00	.58^c^	.33^c^	.33^c^	.41^c^	.21	.14	.13
5. Intention	.54^c^	.56^c^	.02	.59^c^	1.00	.31^c^	.39^c^	.38^c^	.23^a^	.02	.11
6. Planning						1.00	.40^c^	.41^c^	.15	.35^b^	.20^a^
7. Treatment self-efficacy							1.00	.39^c^	.27^b^	.16	.10
8. Subjective engagement frequency								1.00	.37^c^	.33^b^	.19
9. Subjective engagement minutes									1.00	.05	.15
10. Objective engagement pages										1.00	.55^b^
11. Objective engagement minutes											1.00
Mean (SD)	24.83 (19.05)	32.67 (5.75)	28.54 (8.56)	17.67 (6.42)	17.51 (4.78)	32.54 (3.50)	24.53 (8.39)	7.85 (4.01)	75.61 (91.60)	81.77 (70.59)	54.16 (83.79)

^a^*P*<.05.

^b^*P*<.01.

^e^*P*<.001.

### Motivational Phase Model

To test the motivational phase, an SEM was analyzed using T1 participants (N=440). Missing data for all items were 0.05% for T1, 1.24% for T2, and 1.39% for T3. Missing data were imputed with maximum likelihood procedure using AMOS v.24. Additionally, we performed a Little’s missing completely at random (MCAR) test, a stricter criterion than missing at random. A Little’s MCAR test with sex and employment as reference variables showed missing data were MCAR for pretreatment self-efficacy items, χ^2^_7_=6.6, *P*=.48, and perceived need items, χ^2^_15_=10.9, *P*=.76. Items were not MCAR for PCL-5, χ^2^_55_=76.6, *P*=.03; outcome expectations, χ^2^_9_=28.2, *P*=.001; or intention, χ^2^_4_=10.0, *P*=.04. However, for each of these measures, less than 0.06% of the items were missing, so all items were imputed together.

The original independence SEM yielded a poor fit with χ^2^_6_(N=440)=245.1, *P*<.001, CFI=0.526, TLI=0.210, and RMSEA=0.315 (90% CI 0.282-0.349). Modification indices suggested that correlating the measurement variables of PCL-5 scores and perceived need, outcome expectations and perceived need, and outcome expectations and pretreatment self-efficacy would improve overall model fit. These correlated errors were included in the final motivational SEM, producing an excellent fit, χ^2^_2_ (N=440)=5.0, *P*=.08, CFI=0.995, TLI=0.974, RMSEA=0.058 (90% CI 0.000-0.124) that explained 48% of the variance. Figure 1 shows the T1 motivational model. In this model, pretreatment self-efficacy (beta=.13, *P*<.001), outcome expectations (beta=.36, *P*<.001), perceived need (beta=.32, *P*<.001), and PCL-5 (beta=.21, *P*<.001) were significant predictors of intention and indicated support for the motivational model hypotheses.

### Volitional Phase Model

A moderated mediation analysis was performed to test the volitional phase with completers (N=115). We handled missing data using the full information maximum likelihood method. The assumption of full information maximum likelihood estimation is that missing data must be at least missing at random to have reliable outcomes. A Little’s MCAR test with sex and employment status as reference points showed that missing data were MCAR, χ^2^_13_=5.6, *P*=.96. The bootstrap CIs revealed a conditional mediation effect of T2 planning on T3 engagement moderated by T2 treatment self-efficacy at low levels of treatment self-efficacy (−1 SD; B=0.89; 95% CI, 0.143-2.605). The conditional indirect effect was nonsignificant at high levels of treatment self-efficacy (+1 SD; B=0.49; 95% CI, −0.020 to 2.099). In the overall model, the direct effect of intention on planning (beta=.21, *P*=.008) was significant, and the direct effect of planning on engagement (beta=.45, *P*=.06) was approaching significance. The direct effect of intention on engagement (beta=.26, *P*=.11) and the interaction effect between planning and treatment self-efficacy on engagement (beta=−.15, *P*=.37) were not significant. These results suggest that for those with lower treatment self-efficacy, T2 planning increased as T1 intention increased, which further enhanced T3 engagement.

Next, to examine whether the variables that were significantly different between the completers of T3 and dropouts affected the results, these variables were included in the model. The baseline PTSD was included in the motivational phase; thus, we did not include it in this analysis. Age and trauma frequency were entered in the model as covariates for engagement. Results were consistent with the results without the covariates. The conditional indirect effect was significant at low levels of treatment self-efficacy (−1 SD; B=0.90; 95% CI, 0.124-2.330). The conditional indirect effect was not significant at high levels of treatment self-efficacy (+1 SD; B=0.47; 95% CI, −0.043 to 1.751).

## Discussion

### Principal Findings

The aim of this study was to examine the associations between motivational and volitional predictors of engagement with an eHealth intervention for trauma recovery. Previous engagement research focused primarily on a-theoretical approaches such as user characteristics and interventions features. These approaches are heavily tied to individual attributes or unique aspects of the eHealth intervention, and few offer general theoretical frameworks for understanding the process of engagement. Thus, no clear model exists to explain what factors influence engagement in eHealth interventions. To the best of our knowledge, this is the first study to focus on the psychological process of eHealth intervention engagement using the theoretical frameworks of social cognitive theory and the HAPA.

#### Motivational Model

The motivational component of the HAPA model indicates individual intention to utilize an eHealth intervention for trauma is significantly related to outcome expectations, pretreatment self-efficacy, perceived need, and PTSD symptom severity. As hypothesized, higher baseline levels of pretreatment self-efficacy (beta=.13) and outcome expectancy (beta=.36) predict greater intention to engage. These results support the findings of previous studies that used the HAPA model for predicting engagement with other health behaviors such as physical activity [38], breast self-examination, and rehabilitation participation [40,64].

In line with previous mental health research [42], this study indicates that higher baseline levels of perceived need are important in predicting greater intention (beta=.32). This study measured the perceived need for a coping support intervention, whereas previous HAPA studies measured a related construct of perceived risk of developing a disease or disorder [64]. Perception of need rather than risk proved to be an important consideration when examining intervention vs prevention behaviors.

Higher baseline PTSD symptoms also predicted greater intention (beta=.21). Research suggests that PTSD symptomatology is one of the determinants of the intention to seek help [27]. However, the original HAPA model [20] did not include symptoms as a predictor of intention. This is likely because of the original model being used to explain physical health behaviors rather than mental health-related behaviors. This also highlights the potential differences in motivational factors between prevention programs and coping support (or symptom relief) interventions. Future studies should examine the differences in the motivational HAPA predictors for symptom-targeted interventions vs prevention programs.

Our results extend the HAPA motivational model suggesting that symptom severity (eg, PTSD) may be an important factor in understanding intention to utilize an eHealth program. These findings indicate that models such as HAPA may need to consider symptoms of physical or mental illness in understanding motivational factors related to intention. Though higher symptoms predicted greater intention to use the intervention, previous studies found higher baseline symptoms associated with lower usage [47]. This may suggest a nonlinear relationship between baseline symptoms and eHealth usage (ie, curvilinear). However, this has yet to be investigated.

#### Volitional Model

The volitional section of the HAPA model suggests that the role of intention on engagement was differential relative to the individual perceptions of treatment self-efficacy. High intentions are associated with higher levels of planning, yet this relationship is relative to the level of perceived treatment self-efficacy. Planning promoted greater engagement only for individuals with low treatment self-efficacy (B=0.89). Consistent with Schwarzer [50], intenders are motivated to change but often do not act because they may lack the right skills to translate their intention into action. In support of this, our study suggests that those intenders who do not have high confidence in their ability to continue to use a trauma recovery eHealth intervention employ self-regulatory strategies such as planning to facilitate engagement with the intervention. This has important implications for eHealth intervention utilization and treatment development.

### Clinical Implications

Improving engagement with therapy, whether in-person or online, can potentially lead to improved therapeutic outcomes. By understanding the impact of phase-specific self-efficacy, perceived need, and outcome expectations, interventions can be designed to enhance these perceptions, which in turn could lead to improved engagement. Specifically, these results suggest that communicating the expected outcomes of an intervention could have a significant impact on initial engagement. For those who have low perceived need and high PTSD symptoms, motivational enhancement to increase perceived need before treatment may lead to improved engagement [65]. Furthermore, for some individuals, including planning in intervention strategies may also improve treatment engagement. These, in turn, can potentially lead to decreased distress symptoms as intervention engagement is one of the most consistent predictors of positive outcomes [66].

Collectively our findings provide a new way to approach our understanding of engagement with eHealth interventions for trauma and eHealth more generally. Future studies should examine additional mediators and moderators to engagement. For example, recent HAPA related research has found perceived social support and self-regulation (ie, motivation and willpower) to also mediate the intention-behavioral gap for physical activity uptake [37].

### Limitations

Although the motivational phase has a high amount of explained variance (48%), it might be because of the cross-sectional analysis. Future studies should examine this phase longitudinally. Whereas our 2-week study examined the pretreatment and treatment phases of the behavioral change process, the HAPA model typically is also applied to the maintenance and recovery phases of health behavior change. These phases may not apply to engaging in an eHealth intervention to manage psychological distress. However, it might be interesting to conceptualize the processes that would bring a person back to an eHealth intervention following an upsurge in symptoms. Individual perceptions of optimism or self-efficacy in managing these challenges, including returning to an eHealth program, are important to consider. This is akin to optimistic beliefs about one’s ability to deal with barriers that arise while maintaining the behavioral change and recovery self-efficacy associated with one’s conviction to get back on track after being derailed [40]. Additionally, our study investigated action planning (eg, when, where, and how) and did not consider coping planning (ie, how one will cope with obstacles). Past research has revealed differential effects of the two planning processes on the translation of intention to action [38]. Future studies should consider additional phases and examine these planning processes separately.

It should be noted that our research design did not allow us to compare the interaction of the HAPA model factors with important aspects of different eHealth trauma interventions (ie, active comparison group). Future studies will need to focus on deconstructing critical intervention components for different eHealth approaches in relation to the HAPA factors (eg, outcome expectations and perceived treatment self-efficacy).

Another limitation of this study was the high dropout attrition rate over the course of the three measurements. Of the 440 who met criteria for the study, only 115 completed the T3 survey. This attrition rate of almost 73.9% was not unusual for eHealth interventions [22] but did affect the quantitative methods used to test the overall HAPA model. Instead of looking at the model as a whole, two separate analyses were performed for the motivational and volitional phases, an approach that is clearly not optimal. A single analysis would require imputing 73.9% of the missing data. We argue this would compromise the accuracy of the results [67]. Thus, we decided to run two separate analyses. These separate models eliminated the possibility of examining the effects of the motivational predictors on the volitional phase of engagement. A further investigation into the relationship between demographic, mental health histories, and social cognitive predictors for noncompleters vs completers may reveal valuable engagement related information as well.

Finally, and perhaps most importantly, the conceptualization of engagement needs further examination. Our measures of engagement focused on frequency and duration. Additional measures of engagement (sometimes referred to as measures of adherence) can include number of log-ins, number of exercises completed, and the number of days used [68]. Interestingly, the subjective measures rather than the objective measures were correlated with the HAPA predictors. This lends support to other research that found engagement to be a complex, multi-faceted experience that cannot be reduced to the patient's interactions with the intervention [53,69]. Our engagement conceptualization did not take into consideration potency, uptake, and user experience [70]. Adherence and dose, which are often defined in terms of predefined expected intensity of usage [45], require in-depth usage analysis of the eHealth intervention and associated outcomes to understand how much usage is needed for effectiveness. A recent study found more than a quarter of their participants used the intervention once (ie, “one-hit-wonder”) because they could get the help they needed, suggesting intervention dosage must be considered when evaluating engagement [71]. Though we included a subjective measure of usage, other perceptions such as affect, attention, and interest were not measured [53,69]. Usability metrics that assess user satisfaction with the aesthetics of an eHealth intervention in terms of its layout, navigation, and content can also influence total engagement [72]. Future studies should evaluate other constructs related to engagement including affect, interest, dosage, and usability metrics. Finally, our study did not consider missing objective engagement data (those who failed to create an account). This type of missingness is not a random, and failure to consider it can lead to biased results [73].

### Conclusions

In conclusion, this study found support for the utilization of the HAPA model for understanding predictors of engagement with an eHealth intervention for trauma recovery. Results of this study help to clarify what makes eHealth interventions work [5]. This novel theoretical approach to eHealth intervention engagement research can be applied to other types of interventions in a variety of domains. The results of this study extended the HAPA model for health behaviors to include additional predictors of engagement. Perceived need, outcome expectations, pretreatment self-efficacy, and PTSD symptoms were all found to be significant positive predictors of intention; and planning mediated the translation of intention into engagement for those with low treatment self-efficacy. This study offers preliminary information suggesting possible differences in social cognitive predictors for coping support programs vs preventive interventions. Future research applying the extended HAPA model of engagement with other eHealth trauma interventions including mobile apps may offer a generalization of our findings.

## Abbreviations

CFI: comparative fit index
DSM-5: Diagnostic and Statistical Manual of Mental Disorders, Fifth Edition
HAPA: health action process approach
MCAR: missing completely at random
MTR: My Trauma Recovery
PCL-5: Posttraumatic Stress Disorder Checklist for Diagnostic and Statistical Manual of Mental Disorders, Fifth Edition
PTSD: posttraumatic stress disorder
RMSEA: root mean square error of approximation
SEM: structural equation model
T: time
TLI: Tucker-Lewis index
UCCS: University of Colorado at Colorado Springs
